# Supplementary material for: Metabolomics-Based Profiling via a Chemometric Approach to Investigate the Antidiabetic Property of Different Parts and Origins of Pistacia lentiscus L
Source: Metabolites. 2023 Feb 14;13(2):275. doi: 10.3390/metabo13020275 (PMC9960292; doi:10.3390/metabo13020275)
Supplement: Supplementary file 1 [file metabolites-13-00275-s001.zip › metabolites-2137098-supplementary.pdf]

metabolites

MDPI

Article

Metabolomics-based profiling via a chemometric approach to investigate the antidiabetic property of different parts and origins of *Pistacia lentiscus* L.

Chabha SEHAKI[1,2], Roland MOLINIE[1], David MATHIRON[3], Jean-Xavier FONTAINE[1], Nathalie JULLIAN[1], Fadila AYATI[2], Farida FERNANE[2], Eric GONTIER[1,*]

[1]BIOPI-UPJV Laboratory UMRT BioEcoAgro INRAE1158, SFR Condorcet FR CNRS 3417, UFR of Sciences, University of Picardie Jules Verne, 33 Rue Saint Leu, 80000 Amiens, France
[2]Laboratory of Natural Resources, University Mouloud Mammeri of Tizi-Ouzou, Tizi-Ouzou 15000, Algeria
[3]Analytical platform, UFR of Sciences, University of Picardie Jules Verne, 33 Rue Saint Leu, 80000 Amiens, France
* Correspondence:eric.gontier@u-picardie.fr; Tel: +00(33)6 59 21 81 08

Supplementary Materials: The following supporting information can be downloaded at: www.mdpi.com/xxx/s1, **Figure S1**: Boxplot showing the α-glucosidase inhibitory effect of lentisk leaves, stems barks and fruits from the mountain and littoral; **Figure S2**: Contribution of the two blocks (neg and pos) to the constitution of the OPLS model; **Table S1**: Weather data collected between 1999 and 2019 at AitIrane, Algeria; **Table S2**: Weather data collected between 1999 and 2019 at Tigzirt, Algeria. **Table S3**: Metabolomic data (OPLS, matrix-negative ionization); **Table S4**: Metabolomic data (OPLS, matrix-positive ionization); **Table S5**: UHPLC-ESI-HRMS data results and OPLS coordinates; **Table S6**: Phenolic compounds identified in *P. lentiscus* extracts by UHPLC-ESI-HRMS in the positive mode.

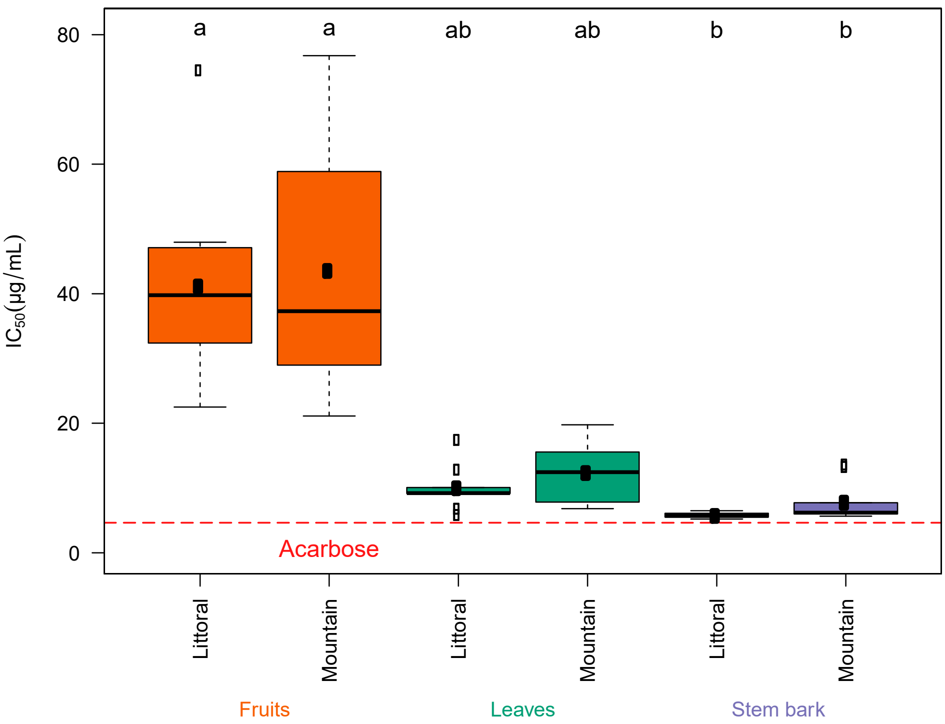

Figure S1. Boxplot showing the α-glucosidase inhibitory effect of lentisk leaves, stems barks and fruits from the mountain and littoral.

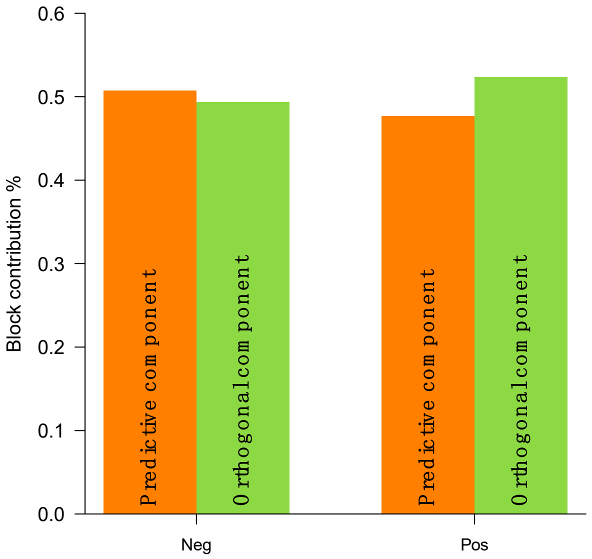

Figure S2. Contribution of the two blocks (neg and pos) to the constitution of the OPLS model.

Table S1. Weather data collected between 1999 and 2019 at Ait Irane, Algeria
(from https://fr.climate-data.org/afrique/algerie/tizi-ouzou/ait-irane-693594/)

	January	February	March	April	May	June	July	August	September	October	November	December
Mean temperature (°C)	5.2	5.3	8.5	11.6	15.6	20.8	24.8	24.5	20	15.9	9.4	6.2
Mean minimum temperature (°C)	0.8	0.5	3.1	5.8	9.4	13.9	17.5	17.8	14.5	10.7	5.2	2.1
Maximum temperature (°C)	10.4	10.7	14.3	17.5	21.6	27.4	31.9	31.6	26.3	21.9	14.5	11.2
Precipitation (mm)	88	78	84	86	74	21	8	19	53	60	81	82
Humidity (%)	79	77	73	70	67	55	45	47	59	64	75	79
Rainy days (jree)	9	8	8	8	7	3	2	4	7	7	9	8
Hours of sunshine (h)	5.7	6.4	7.6	9	10.3	12.3	12.6	11.7	9.9	8.4	6.4	5.7

Table S2. Weather data collected between 1999 and 2019 at Tigzirt, Algeria.
(from https://fr.climate-data.org/afrique/algerie/tizi-ouzou/tigzirt-321206/)

	January	February	March	April	May	June	July	August	September	October	November	December
Mean temperature (°C)	10.2	10	12.1	14.2	17.1	21.1	24.4	25	22.3	19.3	14.1	11.3
Mean minimum temperature (°C)	7.7	7.4	9.3	11.3	14.1	17.9	21.2	22	19.6	16.5	11.7	8.9
Maximum temperature (°C)	12.8	12.7	14.8	16.8	19.7	23.9	27.3	27.9	25	22.2	16.8	13.9
Precipitation (mm)	142	107	86	68	51	9	2	6	36	73	140	146
Humidity(%)	75	74	75	77	77	72	69	69	71	71	73	74
Rainy days (jree)	10	8	7	6	4	1	0	1	4	6	10	10
Hours of sunshine (h)	7	7.8	9	10.2	11.1	12.2	12.3	11.3	10.1	9.1	7.6	7

Table S6. Phenolic compounds identified in *Pistacia lentiscus* extracts by UHPLC-ESI-HRMS in the positive mode.

N° Metabolite	RT (min)	m/z (obs) $(M-H)^-$	Error (ppm)	$HDMS^E$ fragment ions (Intensity, %)	Molecular formula	Suggested compound
1	0.45	611.1391	-0.59	425.08572 (42.86),299.05412(66.25), 263.05419(43.44), 175.03792 (20.42)	$C_{30}H_{26}O_{14}$	Epigallocatechin(4a>8)epigallocatechin
3	0.56	763.1505	-0.29	425.08596(28.64),299.05476(2 3.31),	$C_{37}H_{30}O_{18}$	(Epi)gallocatechin-3′-Ogalloyl-(epi)gallocatechin
4	0.61	611.1383	-1.99	425.08572 (42.86), 299.0542 (66.25), 263.05419 (43.44)	$C_{30}H_{26}O_{14}$	Epigallocatechin(4a->8)epigallocate-chin (isomer)
5	0.72	763.1504	0.31	/	$C_{37}H_{30}O_{18}$	(Epi)gallocatechin-3′-Ogalloyl-(epi)gallocatechin (isomer)
